# Evolutionary history of the UCP gene family: gene duplication and selection

**DOI:** 10.1186/1471-2148-8-306

**Published:** 2008-11-03

**Authors:** Joseph Hughes, Francois Criscuolo

**Affiliations:** 1 University of Glasgow, IBLS/DEEB, Graham Kerr Building, Glasgow, G12 8QQ, UK; 2 Institut Pluridisciplinaire Hubert Curien, Departement Ecologie, Physiologie et Ethologie, UMR 7178-CNRS, 23 rue Becquerel, 67087 Strasbourg Cedex 2, France

## Abstract

**Background:**

The uncoupling protein (UCP) genes belong to the superfamily of electron transport carriers of the mitochondrial inner membrane. Members of the uncoupling protein family are involved in thermogenesis and determining the functional evolution of UCP genes is important to understand the evolution of thermo-regulation in vertebrates.

**Results:**

Sequence similarity searches of genome and scaffold data identified homologues of UCP in eutherians, teleosts and the first squamates uncoupling proteins. Phylogenetic analysis was used to characterize the family evolutionary history by identifying two duplications early in vertebrate evolution and two losses in the avian lineage (excluding duplications within a species, excluding the losses due to incompletely sequenced taxa and excluding the losses and duplications inferred through mismatch of species and gene trees). Estimates of synonymous and nonsynonymous substitution rates (dN/dS) and more complex branch and site models suggest that the duplication events were not associated with positive Darwinian selection and that the UCP is constrained by strong purifying selection except for a single site which has undergone positive Darwinian selection, demonstrating that the UCP gene family must be highly conserved.

**Conclusion:**

We present a phylogeny describing the evolutionary history of the UCP gene family and show that the genes have evolved through duplications followed by purifying selection except for a single site in the mitochondrial matrix between the 5^th ^and 6^th ^α-helices which has undergone positive selection.

## Background

The mitochondrion is the main intracellular site of energy production and is the evolutionary response to the main challenge that living organisms have to face: gaining energy from their environments to sustain their biological functions. The mitochondrial production of ATP is realised by the combination of the phosphorylation of ADP into ATP with an efficient chain of redox reactions, resulting in the so-called oxidative phosphorylation. However, these two processes are not always efficiently coupled, and one reason is the presence in the inner membrane of a family of mitochondrial transporters: the uncoupling proteins (UCP, [1]). UCP1 was first discovered and cloned in 1986 [2] and is involved in the non-shivering thermogenesis (NST) activity of rodent's brown adipose tissue (BAT, [3]). Since then, the discovery of UCP genes has grown rapidly, UCP1 homologues being found across mammalian species (UCP2, UCP3, [4,5]) but also in other eukaryotes from plants to animals [6-8]. Most of the recent attention has been devoted to the evolutionary history of UCP1 since the discovery of UCP1 in ectotherm organisms like teleost fish [9] and amphibians [10]. The fact that organisms that do not show NST possess and express UCP1 raised the question of the exact evolutionary history of UCP1 and of its link with the apparition of thermoregulation. This observation has stimulated an increasing number of phylogenetic studies on UCP [10-15] to determine the origin of the physiological particularity (cold-induced thermogenesis in BAT) in the mammalian lineage [13].

UCP1 and its close homologues (UCP2 and UCP3) are thought to differ in the nature of their uncoupling activity [16,17] and their potential physiological roles (see [18]). Indeed, a rapid overview of the data collected on UCP1, 2 and 3 highlights how these proteins may be different. First, while UCP1 tissue expression is localized (and abundant) to BAT, UCP2 is expressed (in smaller quantities) in a wider range of cell types (like immune or pancreatic β-cells) and UCP3 is mainly present in skeletal muscle ([4,5], see [19]). Also, the physiological role of UCP1 is restricted to thermogenesis, which is unlikely to be the case for UCP2 and 3 as shown by their respective knock-out models [20,21]. UCP2 and 3 have been involved in a number of postulated functions in energy regulation, including regulation of insulin secretion [22] or reactive oxygen species production and control of the immune response [20,23,24]. However, accurate data on the mitochondrial activity of UCP2 and UCP3 are still lacking to determine the exact nature of their biological activity [17,25]. Therefore, despite the high sequence identity shared by UCP1, 2 and 3 (close to 60% in humans and mice), punctual amino acid replacement at key structural domains of the respective proteins may have evolved to allow functional specificity to take place. Interestingly, mutagenesis experiments have shown that single amino-acid replacement in UCP1 protein may change its proton permeability (nature of the mitochondrial transport), its sensibility to fatty acid activation or nucleotide inhibition (regulation of the activity, [26]), or its transmembrane structure [27]. The next step in the understanding of the biology of UCP is to determine whether the evolution of UCP genes and protein sequences may have been subjected to different selective pressures after duplication.

Single copy genes are thought to evolve conservatively because of strong negative selective pressure. Gene duplications produce a redundant gene copy and thus release one or both copies from negative selection pressure. There are a number of models for the fate of gene duplicates, the two most prominent of which are neofunctionilization and subfunctionalisation. Thus, duplications are thought to be an important precursor of functional divergence. The increased availability of UCP sequences in the public databases allows the study of the molecular evolution of the UCP gene family and the evaluation of selection following duplication events. In the present study, we will determine (1) the evolutionary history of the UCP gene family, (2) evaluate the changes in selection pressures following duplications, and (3) identify sites under positive Darwinian selection.

## Results

### Sequence similarity searches and multiple alignment

Two lizard sequences from *Anolis carolinensis *were identified during similarity searches with high similarity to UCP2 and UCP3. Homologues of UCP1 were not found in the lizard scaffold genome. Table 1 outlines the sequences (protein and DNA) used in the phylogenetic analyses. It should be noted that additional UCP genes for eutherians and teleosts were identified. Inclusion of these did not improve the reliability of the phylogeny, and as the aim of this study was to determine the evolutionary history of the UCP gene family, only representatives from the major vertebrate clades were included.

**Table 1:** List of species and accession numbers for protein and DNA sequences

	Protein		DNA	
***Species name***	***Name***	***Accession***	***Name***	***Accession***

*Arabidopsis thaliana*	Aratha21593775	AAM65742.1	Atha_UCP	NM_115271.4
*Zea mays*	Zeamay19401698	AAL87666.1		
*Solanum tuberosum*			Stu_UCP	Y11220.1
*Anopheles gambiae*	Anoga11676	AGAP011676-PA (b)	Aga	XM_552584.3
*Apis mellifera*	Apime66501089	XP_394267.2	UCP1Ame	XM_394267
*Strongylocentrotus purpuratus*	Strpur115969038	XP_001185598.1		
*Ciona intestinalis*	Cint23999	ENSCINP00000023999 (b)	UCP_Cint	AK113254.1
*Homo sapiens*	HosapUCP1	P25874	UCP1Hsa	NM_021833
*Mus musculus*	MusmuUCP1	P12242.2	UCP1Mmu	NM_009463
*Bos taurus*	BotauUCP1	P10861.2	UCP1Bta	XM_616977
*Sminthopsis crassicaudata*	SmcraUCP1	ABR32188.1	UCP1Scra	EF622232
*Monodelphis domestica*	Modom126331519	XP_001377555.1	UCP1Mdo1	XM_001377518
*Ornithorhynchus anatinus*	Oana149635652	XP_001512700.1	UCP1Oana	XM_001512650
*Xenopus tropicalis*	Xetro166157878	NP_001107354.1	Xentrop	NM_001113882.1
*Xenopus laevis*	Xelae147898993	NP_001088647.1	UCP1s429	BC086297
*Danio rerio*	DareUCP4	NP_955817.1	UCP4Dre	BC075906
*Danio rerio*	DareUCP3	AAQ97861.1	UCP3Dre	AY398428
*Cyprinus carpio*	CypcaUCP1	AAS10175.2	UCP1Cca	AY461434
*Tetraodon nigroviridis*	Tenig9630	ENSTNIP00000009630 (b)		
*Takifugu rubripes*	TakrubUCP1	ENSTRUP00000033443 (b)		
*Homo sapiens*	HosapUCP2	P55851.1	UCP2Hsa	NM_003355
*Mus musculus*	MumusUCP2	P70406.1	UCP2Mmu	NM_011671
*Bos taurus*	BotauUCP2	XP_614452.1	Bosta_UCP2	NM_001033611.1
*Antechinus flavipes*	AnflaUCP2	AAP44414.1	UCP2Afl	AY233003
*Sminthopsis macroura*	SmmacUCP2	AAP45779.1	UCP2Sma	AY232996
*Monodelphis domestica*	ModomUCP2	XP_001362966.1	UCP2Mdo	XM_001362929
*Ornithorhynchus anatinus*	OanaUCP2	XP_001512584.1	UCP2Oana	XM_001512534
*Anolis carolinensis (a)*	Anca1518	scaffold_1518:59062–63752	UCP2_anca	scaffold_1518:59062–63752
*Cyclorana alboguttata*	CycalbUCP2	ABK96864	Cycalb_UCP2	EF065613.1
*Xenopus laevis*	XelaeUCP2	AAH44682.1	UCPs1234	NM_001086754
*Xenopus tropicalis*	XetroUCP2	AAH63352.1	UCPxtr	NM_203848
*Cyprinus carpo*	CypcaUCP2	Q9W725.1	Cypca_UCP2	AJ243486.1
*Danio rerio*	DareUCP2	CAB46268.1	UCP2Dre	AJ243250
*Tetraodon nigroviridis*	Tetnig47222581	CAG02946.1		
*Takifugu rubripes*	TakrubUCP2	ENSTRUP00000037074 (b)		
*Zoarces viviparus*	ZovivAAT99594	AAT99594		
*Homo sapiens*	HosapUCP3	P55916.1	UCP3Hsa	NM_003356
*Mus musculus*	MusmuUCP3	P56501.1	UCP3Mmu	NM_009464
*Bos taurus*	BotauUCP3	O77792.1	UCP3Bta	NM_174210
*Gallus gallus*	GalgaUCP3	NP_989438.1	UCPGga	AB088685
*Meleagris gallopavo*	Melgal16755900	AAL28138		
*Eupetomena macroura*	Eumac13259162	AAK16829.1	UCPEma	AF255729
*Antechinus flavipes*	AnflaUCP3	AAS45212.1	UCP3Afl	AY519198
*Monodelphis domestica*	ModomUCP3	XP_001368096.1	UCP3Mdo	XM_001368059
*Ornithorhynchus anatinus*	OanaUCP3	XP_001512822.1	UCP3Oana	XM_001512772
*Anolis carolinensis (a)*	Anca1149	scaffold_1149:20424–36291	Lizard	scaffold_1149:20424–36291
*Xenopus tropicalis*	XentrUCP3	e_gw1.1014.45.1 *		
*Danio rerio*	Dare50936	ENSDARP00000050936 (b)		
*Petromyzon marinus*	Pemar51797123	CO548809.1	SeaLamprey	CO548809.1
*Lethenteron japonicum*	Lejap149930881	ABR45662.1	Letjap_UCP	EF644490.1
*Takifugu rubripes*	TakruUCP3	ENSTRUP00000037001 (b)		

(a) sequence obtained from the February 2007 draft assembly (Broad Institute AnoCar (1.0)) produced by the Broad Institute at MIT and Harvard, (b) sequences obtained from Ensembl, * sequences from JGI.

### Phylogeny of the UCP gene family

**Figure 1:**
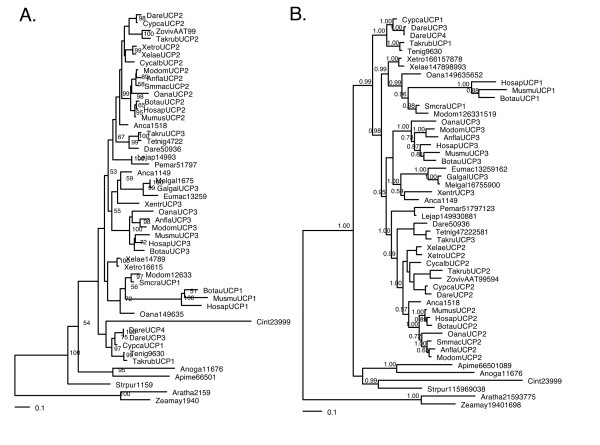
**Phylogenetic relationships of proteins within the UCP family**. (A) Maximum likelihood method with bootstrap support (500 pseudo-replicates) above 50% shown at the nodes (likelihood of -7746.18) and (B) Bayesian inference with posterior probability shown at the nodes (likelihood of -8900.97). All trees were rooted with the plant UCP proteins.

**Figure 2:**
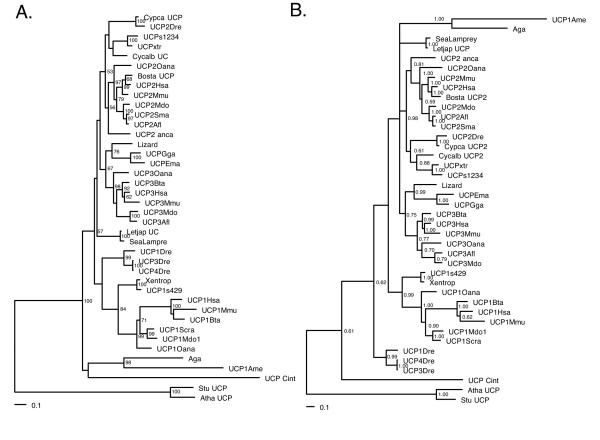
**Phylogenetic relationships of DNA sequences**. (A) Maximum likelihood method with bootstrap support (500 pseudo-replicates) above 50% shown at the nodes ((likelihood of -16059.84) and (B) Bayesian inference with posterior probability shown at the nodes (likelihood of -15825.16). All trees were rooted with the plant sequences.

The alignments were used to construct phylogenetic trees with maximum likelihood (ML) and Bayesian inference (BI). The different reconstruction methods provided poor support for basal nodes using the protein alignment (Figure 1). The DNA alignment showed support for the UCP1, UCP2 and UCP3 clades, in particular when fewer distantly related outgroups are used perhaps as a consequence of systematic error (Figure 2 and see additional material 1). The different reconstruction methods provided slightly different topologies. Most relationships could be resolved with confidence dividing the gene family into strongly supported clusters in most tree reconstructions. UCP2 and UCP3 genes are sister clusters and the avian UCP gene is grouped within the UCP3 cluster.

The 2 different protein trees were reconciled against a species tree using GeneTree. The protein ML topology required 14 duplications and 47 losses and the BI 15 duplications and 51 losses. The high number of duplications and losses is a result of the basal topology of the gene tree and a number of incongruences between the gene and species trees. However, in the ML protein phylogenies, the basal relationships have low bootstrap supports. Using the DNA phylogeny, the ML tree required less duplications and losses (8 d + 2 l) than the BI tree (12 d + 42 l). The higher number of duplications and losses in the BI reconstruction is mainly a result of duplications inferred through incongruence between the gene and species trees.

**Figure 3:**
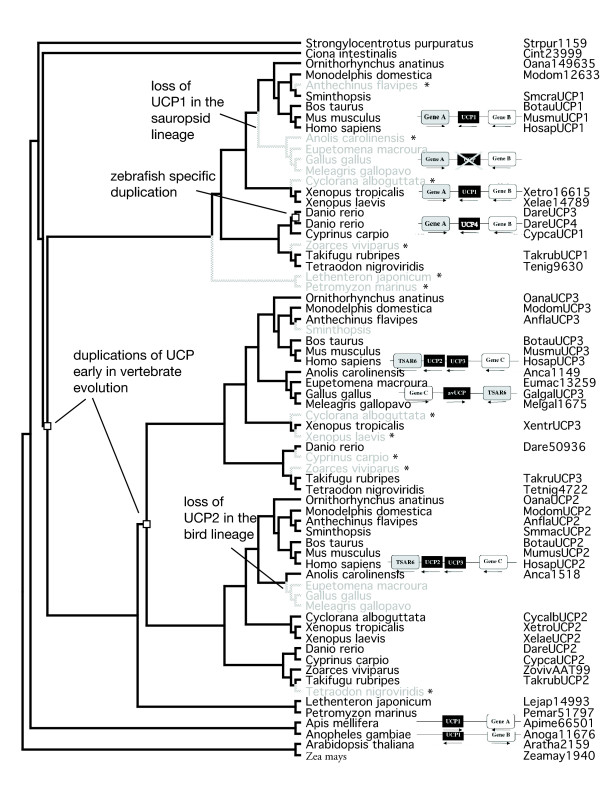
**Reconciled tree for the UCP gene family**. The ML tree of UCP genes was reconciled using GeneTree with a species tree. Squares indicate duplication events, grey lines indicate absent genes, either lost from those species or not yet sequenced. An asterisk represents a postulated loss due to incomplete genome sequences. The schematic gene maps of the conserved syntenic regions of the uncoupling proteins according to the study of Emre et al [10].

If as suggested by the protein phylogenies, the lamprey sequences are sister to the UCP2 clade and the *Takifugu *UCP3 groups within the same clade, then the reconciliation infers 3 duplications (excluding species specific duplications) and 4 losses (excluding losses as a result of incomplete data). However, the results from the DNA phylogenies suggest the lamprey sequences could have diverged before the duplication of UCP2/3. In this case, by removing losses and duplications inferred through mismatch of species and gene trees and losses due to incomplete genome sequences, the most parsimoniously reconciled tree shows 1 zebrafish specific duplication and two major duplications that occurred early in the vertebrate lineage (Fig. 3). One duplication is proposed to have occurred prior to the emergence of teleost fish resulting in two lineages which evolved into UCP1 and UCP2/3 and probably took place early in vertebrate evolution due to the presence of UCP2 in lampreys, although further data are required to confirm the presence of UCP1 and UCP3 in lampreys. A second duplication, also early in vertebrate evolution, resulted in UCP2 and UCP3. Further sequencing of a broader range of ancestral craniata is required to identify a more precise timing for the duplications. Interestingly, UCP2 and UCP1 have been independently lost from the avian lineage but further data are required to confirm the absence of UCP1 in lizards to be able to determine when the loss of UCP1 took place.

### Synonymous and non-synonymous substitution rate estimates

Results using the DNA dataset show that UCP genes are under varying selection pressures (Table 2). Pairwise comparisons of human and mouse orthologs and human and platypus show that UCP1 has higher estimates of dN/dS ratio compared to UCP2 and UCP3 but suggest purifying selection in all three genes. The lower substitution rates for UCP2 and UCP3 shows that they are under strong purifying selection.

**Table 2:** Synonymous (dS) and nonsynonynous (dN) substitution rates for all UCP genes

	human-mouse			human-platypus		
	
	*dN*	*dS*	*dN/dS*	*dN*	*dS*	*dN/dS*
UCP1	0.111	0.713	0.156	0.217	0.963	0.225
UCP2	0.018	0.672	0.027	0.044	1.083	0.041
UCP3	0.033	0.674	0.049	0.062	1.080	0.058

Substitution rates were estimated using Yang and Nielson [53] method as implemented in yn00 in the PAML package.

### Positive selection tests

More sophisticated codon-based substitution models were used to test for branch-specific selection. The model was based on the assumption that selective constraints change following gene duplication. We estimated ω as an average over all sites and branches and the ratio was substantially smaller than 1 (one ratio model ω = 0.07649, Table 3). The one-ratio model was compared with model R2, and the LRT (Table 4) indicated that there is a significant decrease in the rate of non-synonymous substitution following the duplication of UCP1 and UCP2/3 (ω_0 _= 0.095 versus ω_1 _= 0.066). The comparison of model R2 and R3 also showed that there was a significant difference in the selective pressure following the duplication of UCP2 and UCP3. The branch specific model with three distinct rates of substitution (R3), one for each UCP gene, is a significantly better fit than the one-ratio (R0) and two-ratio (R2) models according to the LRT (Table 4). This suggests significantly different selective pressures on UCP1, UCP2 and UCP3. However, none of the parameters estimated indicate positive Darwinian selection.

**Table 3:** Parameter estimates for UCP genes under different branch models, site models and branch-site models

Model	Parameters for branches	Positively selected sites	Likelihood
One-Ratio	ω_0 _= 0.07649	None	-15704.05
Branch specific			
Two-ratios (R2)	ω_0 _= 0.0950	None	-15696.32
	ω_1 _= 0.0660		
Three-ratios (R3)	ω_0 _= 0.0946	None	-15686.87
	ω_1 _= 0.0845		
	ω_2 _= 0.0496		
***Site specific***			
Neutral (M1)	ω_0 _= 0.0657, ρ_0 _= 0.92529	Not allowed	-15586.80
	ω_1 _= 1, ρ_1 _= 0.07471		
Selection (M2)	ω_0 _= 0.06571, ρ_0 _= 0.9253		-15586.80
	ω_1 _= 1, ρ_1 _= 0.07470		
	ω_2 _= **1.266**, ρ_2 _= 0.00000		
Discrete (M3)	ω_0 _= 0.1109, ρ_0 _= 0.49068		-15244.05
(K = 3)	ω_1 _= 0.10086, ρ_1 _= 0.38265		
	ω_2 _= 0.32064, ρ_2 _= 0.12668		
Beta (M7)	ρ = 0.50769 q = 4.86274		-15243.99
Beta&ω (M8)	ρ_0 _= 0.99, p = 0.53223	224 K (P = 0.914)	-15233.43
	q = 5.57248, ρ_1 _= 0.00373, ω_1 _= **1.69524**		
***Branch-Site***			
Model A	ρ_0 _= 0.9155, ρ_1 _= 0.04732,	In the foreground lineage:	-15563.81
	ρ_2a _= 0.03529, ρ_2b _= 0.00182	180 H (P = 0.953), 220 L (P = 0.997), 235 M (P = 0.969)	
	ω_2 _= 0.06259		
			
Model B	ρ_0 _= 0.49203, ρ_1 _= 0.43366,	In foreground lineage:	-15264.48
	ρ_2a _= 0.03950, ρ_2b _= 0.03481	No significant sites	
	ω_0 _= 0.012, ω_1 _= 0.12812, ω_2 _= 0.52	In the background lineage:	
		no significant site	

The models were implemented in Codeml from PAML. Parameters in bold indicate positive selection.

**Table 4:** Likelihood ratio test statistics (2δ) for the test of model fit

	2δ	df	LRT p
H0		NA	NA
One ratio versus H1	15.4	1	<0.001
One ratio versus H2	34.3	1	<0.001
H1 vs H2	18.9	1	<0.001
			
LRTs of variable w's among sites			
One ratio vs. M3	919.9	4	0
M1 vs M2	0	2	1
M7 vs M8	21.1	2	<0.001

Significant tests are shown in bold.

**Figure 4:**
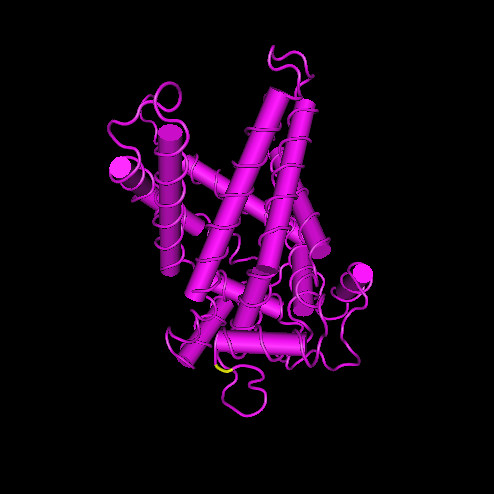
**Three-dimensional representation of the UCP molecule according to the 3D structure of bovine mitochondrial adp-atp carrier **(PDB id: 2c3e). The site under positive selection between the 5^th ^and 6^th ^α-helices is shown in yellow.

**Figure 5:**
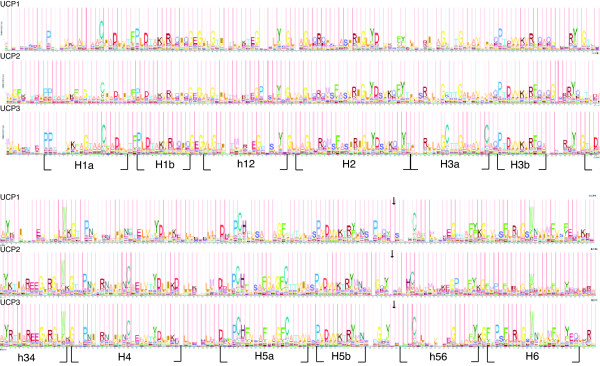
**HMM Logos for UCP1, UCP2 and UCP3**. Comparison of the HMM Logos of UCP1, UCP2 and UCP3 protein alignments (excluding variable regions). The numbering of each α-helix follows the nomenclature used for the ADP/ATP carrier [55]. The site under positive selection in model M8 is indicated with an arrow.

The LRT of the one ratio model with M3 indicates that selective pressure is not uniform among sites (2δ = 919.98, d.f. = 4, p < 0.00001, Table 4). Only the M8 model indicates a site that is evolving under positive Darwinian selection (Table 3). LRTs (Table 4) indicate that model M2 does not fit the data better than M1 whilst it does show that M8 fits the data better than M7, which does not allow for positively selected sites (M1 vs M2: 2δ = 0, d.f. = 2, p = 1.0; M7 versus M8: 2δ = 21.12, d.f. = 2, P < 0.0001). Branch-site models were also applied with UCP1 specified as the foreground branch, however the M8 model was the best fit to the data with a likelihood value of – 15233 (Table 3). This model suggests that the variation in selection pressure is due to the evolution by positive selection of a single site, whilst the remaining sites are under strong purifying selection. According to the alignment of the UCP sequences with the 3D structure of the bovine mitochondrial ADP-ATP carrier (PDB id: 2c3e), the site under positive selection is in the mitochondrial matrix between the 5^th ^and 6^th ^alpha-helix (Fig 4). As illustrated using HMM logos [28] for each UCP gene, the site under positive selection follows a highly conserved (Y) amino acid site present across the whole UCP gene family but the site under positive selection is not conserved within the different UCP groups (Fig 5). The HMM logos also illustrate the high level of sequence conservation in the gene family.

## Discussion

In this study, we have sought to expand upon previous phylogenetic studies [10,15,26] by focusing on the UCP gene families and incorporating sequences identified from completed genomes with a subset of cloned sequences, particularly those from non-mammalian species. This study is the first to include lizard UCP genes. The phylogenetic tree reconstruction of DNA sequences gave well resolved topologies with stronger support values for basal relationships than using the protein data probably as a result of the highly conserved protein sequences. These phylogenies provided a method to infer the evolutionary history of the UCP gene family.

The phylogeny of the UCP genes indicates that UCP1, which is present in plants and Arthropods, is the ancestral UCP as demonstrated in previous studies [10,15,26]. UCP1 then duplicated prior to the divergence of vertebrates. A second duplication of UCP2 and UCP3 also took place early in vertebrate evolution although the exact timing of the event (before or after the divergence of lampreys) requires further genomic data to be gathered. The multiple sequences of UCPs found in the zebrafish, while termed UCP4 and UCP3 are both UCP1 orthologs and should be called UCP1a and UCP1b. UCP4 is syntenic to Gene A and Gene B like other vertebrate UCP1 genes (Figure 3). This could either be a zebrafish specific duplication, or the incomplete sequencing of *Cyprinus carpio *could be hiding an additional paralog and the duplication may be a fish specific genome wide duplications hypothesised to have occurred during fish evolution [29,30]. The latter is probably unlikely due to the lack of duplicates in the complete genome of *Takifugu rubripes*. Importantly, the phylogenetic analyses suggest the independent loss of UCP1 and UCP2 from the avian lineage. The absence of UCP1 in the lizard genome could be attributed to the incompleteness of the genome or could be the result of a loss of UCP1 in the sauropsid lineage.

UCP1 is the only uncoupling protein for which there is a scientific consensus concerning the nature of its physiological function (thermogenesis, [31]). The UCP1 knockout mice are able to maintain their body temperature, but suffer in pronounced cold exposure suggesting that UCP1 is principally involved in short-term adaptation to cold (Enerback et al. 1997). This adaptive evolution probably occurred after the divergence between eutherians and marsupials [13] consistent with the fact that BAT is only found in eutherians. Even though birds are lacking UCP1, they are still able to respond to thermal challenges. The loss of UCP1 and disappearance of BAT in birds is likely due to the concomitant development of physiological adaptations which have replaced BAT function. As evidence, metabolic rate of birds increases in response to cold and body temperature can be maintained [32]. Indeed, induced uncoupling activity in the mitochondria has been found in the skeletal muscle of cold-acclimated birds [33,34] and more recently the implication of UCP3 (avianUCP) has been suggested [35]. These data lead to two non exclusive conclusions. Firstly, birds have evolved other mechanisms of thermoregulation [25] before or after the loss of UCP1 and BAT (e.g.: futile cycle of Ca^2+ ^in bird skeletal muscle or greater adenine nucleotide translocase-catalysed proton conductance, [35,36]). Secondly, a fully demonstrated implication of UCP3 (avianUCP) in skeletal non-shivering thermogenesis in birds would suggest that UCP3, which is not involved in thermoregulation in mammals [21,37], has acquired a new function in birds. In this case, the question is whether avianUCP activity could also compensate for the loss of the ucp2 gene, implicated in mammalian immunity [20] and glucose metabolism [22]. This is an interesting point given the non pathologic high chronic glycemia of birds [38].

The molecular evolution of UCP genes showed that they were under strong purifying selection with a significant change towards stronger purifying selection. UCP1 has the highest dN/dS ratio followed by UCP3 and then UCP2. This strong purifying selection highlights the importance of the function of this highly conserved gene family. Although highly variable regions of the sequence which were difficult to assign as homologous were removed from the analyses, the site models showed that adaptation has appeared at a single site located between the 5^th ^and 6^th ^α-helices. The role of this positively selected site has yet to be determined but the amino acid site (Y) immediately prior to it is highly conserved across mitochondrial carriers as are the transmembrane regions that follow the site. Additionally, Saito et al. [13] found that the two amino acid sites that follow this site are conserved in all eutherian mammal ucp1 genes. Based on studies conducted on UCP1, the region delimited by the 5^th ^and 6^th ^α-helices is close to a site of regulation of UCP1 activity by nucleotides and thus could be implicated in the inhibitory control of UCP1 uncoupling effect [15,26]. This region is also hypothesized to be implicated in the mechanism of transport of protons/free fatty acids [39] in UCP1. However, to date there is a gap in the knowledge of the relationship between amino acid sequence and structure for UCP2 and UCP3, and we are unable to speculate on the particular role of this region in these UCP1 homologues. Unfortunately, shared evolutionary history and molecular selection alone cannot be used as the unique criterion to infer protein function, and the true nature of each UCP gene needs to be determined experimentally and independently. Therefore, this positively selected site may play an important functional role and could represent an interesting target site for future mutagenesis experiment thus facilitating our understanding of the structure-function relationships in UCP genes.

## Conclusion

Genomic data have provided an opportunity to gain a better understanding about the evolution of UCPs using phylogenetic analyses. The UCP gene family phylogeny shows that two duplications took place early in the evolution of vertebrates. Subsequent to these two duplications, UCP1 and UCP2 were lost from the avian lineage independently. However, further genome projects on a greater diversity of evolutionary lineages are required to better understand the gene-duplication history. Evolutionary rate analysis shows purifying selection across branches and sites (except for one single site with site specific positive selection) suggesting that the function of the genes in the UCP gene family has been highly conserved after duplication events and over evolutionary time. By considering the evolutionary history of the UCP gene family we provide insight into which amino acid residues might have undergone positive selection and could be targeted for site-directed mutagenesis. However, the identification of a single site under positive selection requires supporting evidence from further studies with better algorithms for a more credible assessment of site-specific subfamily divergence.

## Methods

### Sequences and sequence similarity searches

Amino acid and nucleotide sequences of UCP gene family members were obtained from GenBank for most species (see Table 1 for accession numbers). The sequences for the lizard (*Anolis carolinensis*) were obtained from the February 2007 draft assembly (Broad Institute AnoCar (1.0)) produced by the Broad Institute at MIT and Harvard [40]. A total of 50 sequences for 27 species were used in the final analyses.

### Multiple sequence alignment and phylogenetic analysis

Fifty protein sequences were aligned using MUSCLE [43] and gaps, which are problematic in phylogenetic analysis, were removed using Gblocks 0.91b [44]. The final protein dataset was 274 amino acids long (Additional material 2). Uncoupling proteins from plants, insects, the sea squirt and the sea urchin were included. Nucleotide sequences were aligned using ClustalX [41] with the default parameters followed by manual alignment in Macclade [42] according to the amino acid translation. Regions before the starting codon were excluded from the analysis as well as regions poorly aligned due to uncertain homology (positions from the first nucleotide of the start codon: 64–66, 142–180, 331–375, 469–504, 931 to end). The final dataset was 810 nucleotides long (Additional material 3).

Phylogenetic trees were reconstructed using maximum likelihood (ML) implemented in PHYML and Bayesian inference (BI) in MrBayes. Phyml v2.4.4 [45] was used with the online web server [46] for maximum likelihood analysis using the GTR+I+G substitution DNA model selected with ModelTest [47] and JTT substitution model selected with ModelGenerator for the protein analyses [48]. The robustness of the trees were assessed by bootstrapping (500 pseudoreplicates) with PHYML. Bayesian analyses were conducted using the same model with MrBayes v3.1.2 [49]. Node support was assessed as the posterior probability from two independent runs each with four chains of 200,000 generations (sampled at intervals of 100 generations with a burn-in of 1000 trees).

### Reconciliation of gene and species trees

Gene trees of the UCP gene family were reconciled with a species tree using GeneTree [50]. GeneTree attempts to resolve the incongruence between the gene and species trees by predicting duplications and losses [50]. The species tree was based on the Tree of Life phylogeny [51] and NCBI taxonomy [52]. The reconciled tree was edited to remove losses and duplications inferred due to mismatches of the species and gene trees.

### Estimation of substitution rates and testing positive selection

Synonymous (dS) and non-synonymous (dN) substitution rates were estimated using the methods of Yang and Nielson [53] as implemented in yn00 in the PAML software [54]. The two trees (ML and BI) were tested separately for positive selection. Using Codeml from PAML the branch specific models, One-ratio (R1) and Two-ratios (R2) were used to detect lineage-specific changes in selective pressure after the duplication events. The site specific models, Neutral (M1), Selection (M2), Discrete (M3) with 3 site classes, Beta (M7) and Beta&ω (M8) were also used to test for individual residues under positive selection. Likelihood ratio tests (LRT) were used to assess their goodness of fit, by comparing a model that does allow for dN/dS>1 against a model that does not (i.e. null model). Therefore, the branch specific LRT was R2 vs R1. The site specific LRTs were M3, M2 and M8 against their respective null models, M0, M1 and M7. Positively selected sites were listed. Because some of the models like M2 and M8 are noted to be prone to the problem of multiple local optima, we ran the program twice, once with a starting omega value <1 and a second time with a value >1. We used the results corresponding to the highest likelihood.

## Authors' contributions

JH performed all sequence and phylogenetic analysis and drafted the methods and result section of the manuscript, FC conceived the study, participated in the design and coordination of the study and drafted the introduction. Both authors drafted the discussion and read and approved the final version.

## Supplementary Material

Additional file 1 **Phylogenies using DNA sequences**. Phylogenies using 29 DNA sequences. (A) Maximum parsimony phylogeny (data analysed in PAUP bootstrapped 1000 times) with bootstrap support above 50 shown at the nodes (tree length of 2763), (B) Maximum likelihood method with bootstrap support (likelihood of -11,992) and (C) Bayesian inference with posterior probability shown at the nodes (likelihood of -11,776). All trees were rooted with *Apis mellifera*. Click here for file

Additional file 2 **Gblocks results**. Sequence alignment of UCP proteins with the selected positions underlined in blue. Click here for file

Additional file 3 **Nexus matrix**. DNA sequence alignment of UCP genes used for building the phylogenies in the nexus format. Click here for file

## References

[B1] Nicholls DG Rial E Brown fat mitochondria Trends Biochem Sci 1984 9 489 491 10.1016/0968-0004(84)90319-0

[B2] Bouillaud F Weissenbach J Ricquier D Complete cDNA-derived amino acid sequence of rat brown fat uncoupling protein J Biol Chem 1986 261 1487 1490 3753702

[B3] Enerback S Jacobsson A Simpson EM Guerra C Yamashita H Harper ME Kozak LP Mice lacking mitochondrial uncoupling protein are cold-sensitive but not obese Nature 1997 387 90 94 10.1038/387090a0 9139827

[B4] Boss O Samec S Paoloni-Giacobino A Rossier C Dulloo A Seydoux J Muzzin P Giacobino JP Uncoupling protein-3: a new member of the mitochondrial carrier family with tissue-specific expression FEBS Lett 1997 408 39 42 10.1016/S0014-5793(97)00384-0 9180264

[B5] Fleury C Neverova M Collins S Raimbault S Champigny O Levi-Meyrueis C Bouillaud F Seldin MF Surwit RS Ricquier D Warden CH Uncoupling protein-2: a novel gene linked to obesity and hyperinsulinemia Nat Genet 1997 15 269 272 10.1038/ng0397-269 9054939

[B6] Laloi M Klein M Riesmeier JW Muller-Rober B Fleury C Bouillaud F Ricquier D A plant cold-induced uncoupling protein Nature 1997 389 135 136 10.1038/38156 9296489

[B7] Raimbault S Dridi S Denjean F Lachuer J Couplan E Bouillaud F Bordas A Duchamp C Taouis M Ricquier D An uncoupling protein homologue putatively involved in facultative muscle thermogenesis in birds Biochem J 2001 353 441 444 10.1042/0264-6021:3530441 11171038 PMC1221587

[B8] Vianna CR Hagen T Zhang CY Bachman E Boss O Gereben B Moriscot AS Lowell BB Bicudo JE Bianco AC Cloning and functional characterization of an uncoupling protein homolog in hummingbirds Physiol Genomics 2001 5 137 145 11285367 10.1152/physiolgenomics.2001.5.3.137

[B9] Jastroch M Wuertz S Kloas W Klingenspor M Uncoupling protein 1 in fish uncovers an ancient evolutionary history of mammalian nonshivering thermogenesis Physiol Genomics 2005 22 150 156 10.1152/physiolgenomics.00070.2005 15886331

[B10] Emre Y Hurtaud C Ricquier D Bouillaud F Hughes J Criscuolo F Avian UCP: the killjoy in the evolution of the mitochondrial uncoupling proteins J Mol Evol 2007 65 392 402 10.1007/s00239-007-9020-1 17909695

[B11] Jastroch M Buckingham JA Helwig M Klingenspor M Brand MD Functional characterisation of UCP1 in the common carp: uncoupling activity in liver mitochondria and cold-induced expression in the brain J Comp Physiol [B] 2007 177 743 752 10.1007/s00360-007-0171-6 17576568

[B12] Jastroch M Withers KW Taudien S Frappell PB Helwig M Fromme T Hirschberg V Heldmaier G McAllan BM Firth BT Marsupial uncoupling protein 1 sheds light on the evolution of mammalian nonshivering thermogenesis Physiol Genomics 2008 32 161 169 17971503 10.1152/physiolgenomics.00183.2007

[B13] Saito S Saito CT Shingai R Adaptive evolution of the uncoupling protein 1 gene contributed to the acquisition of novel nonshivering thermogenesis in ancestral eutherian mammals Gene 2008 408 37 44 10.1016/j.gene.2007.10.018 18023297

[B14] Sokolova IM Sokolov EP Evolution of mitochondrial uncoupling proteins: novel invertebrate UCP homologues suggest early evolutionary divergence of the UCP family FEBS Lett 2005 579 313 317 10.1016/j.febslet.2004.11.103 15642337

[B15] Jimenez-Jimenez J Zardoya R Ledesma A Garcia de Lacoba M Zaragoza P Mar Gonzalez-Barroso M Rial E Evolutionarily distinct residues in the uncoupling protein UCP1 are essential for its characteristic basal proton conductance J Mol Biol 2006 359 1010 1022 10.1016/j.jmb.2006.04.022 16697409

[B16] Couplan E del Mar Gonzalez-Barroso M Alves-Guerra MC Ricquier D Goubern M Bouillaud F No evidence for a basal, retinoic, or superoxide-induced uncoupling activity of the uncoupling protein 2 present in spleen or lung mitochondria J Biol Chem 2002 277 26268 26275 10.1074/jbc.M202535200 12011051

[B17] Mozo J Ferry G Studeny A Pecqueur C Rodriguez M Boutin JA Bouillaud F Expression of UCP3 in CHO cells does not cause uncoupling, but controls mitochondrial activity in the presence of glucose Biochem J 2006 393 431 439 10.1042/BJ20050494 16178820 PMC1383702

[B18] Criscuolo F Gonzalez-Barroso Mdel M Bouillaud F Ricquier D Miroux B Sorci G Mitochondrial uncoupling proteins: new perspectives for evolutionary ecologists Am Nat 2005 166 686 699 10.1086/497439 16475085

[B19] Ricquier D Bouillaud F The uncoupling protein homologues: UCP1, UCP2, UCP3, StUCP and AtUCP Biochem J 2000 345 Pt 2 161 179 10.1042/0264-6021:3450161 10620491 PMC1220743

[B20] Arsenijevic D Onuma H Pecqueur C Raimbault S Manning BS Miroux B Couplan E Alves-Guerra MC Goubern M Surwit R Disruption of the uncoupling protein-2 gene in mice reveals a role in immunity and reactive oxygen species production Nat Genet 2000 26 435 439 10.1038/82565 11101840

[B21] Vidal-Puig AJ Grujic D Zhang CY Hagen T Boss O Ido Y Szczepanik A Wade J Mootha V Cortright R Energy metabolism in uncoupling protein 3 gene knockout mice J Biol Chem 2000 275 16258 16266 10.1074/jbc.M910179199 10748196

[B22] Zhang CY Baffy G Perret P Krauss S Peroni O Grujic D Hagen T Vidal-Puig AJ Boss O Kim YB Uncoupling protein-2 negatively regulates insulin secretion and is a major link between obesity, beta cell dysfunction, and type 2 diabetes Cell 2001 105 745 755 10.1016/S0092-8674(01)00378-6 11440717

[B23] Emre Y Hurtaud C Karaca M Nubel T Zavala F Ricquier D Role of uncoupling protein UCP2 in cell-mediated immunity: how macrophage-mediated insulitis is accelerated in a model of autoimmune diabetes Proc Natl Acad Sci USA 2007 104 19085 19090 10.1073/pnas.0709557104 18006654 PMC2141912

[B24] Emre Y Hurtaud C Nubel T Criscuolo F Ricquier D Cassard-Doulcier AM Mitochondria contribute to LPS-induced MAPK activation via uncoupling protein UCP2 in macrophages Biochem J 2007 402 271 278 10.1042/BJ20061430 17073824 PMC1798432

[B25] Mozo J Emre Y Bouillaud F Ricquier D Criscuolo F Thermoregulation: what role for UCPs in mammals and birds? Biosci Rep 2005 25 227 249 10.1007/s10540-005-2887-4 16283555

[B26] Jimenez-Jimenez J Ledesma A Zaragoza P Gonzalez-Barroso MM Rial E Fatty acid activation of the uncoupling proteins requires the presence of the central matrix loop from UCP1 Biochim Biophys Acta 2006 1757 1292 1296 10.1016/j.bbabio.2006.05.027 16814247

[B27] Gonzalez-Barroso MM Fleury C Levi-Meyrueis C Zaragoza P Bouillaud F Rial E Deletion of amino acids 261–269 in the brown fat uncoupling protein converts the carrier into a pore Biochemistry 1997 36 10930 10935 10.1021/bi971104y 9283084

[B28] Schuster-Bockler B Schultz J Rahmann S HMM Logos for visualization of protein families BMC Bioinformatics 2004 5 7 10.1186/1471-2105-5-7 14736340 PMC341448

[B29] Peer Y Van de Taylor JS Meyer A Are all fishes ancient polyploids? J Struct Funct Genomics 2003 3 65 73 10.1023/A:1022652814749 12836686

[B30] Steinke D Hoegg S Brinkmann H Meyer A Three rounds (1R/2R/3R) of genome duplications and the evolution of the glycolytic pathway in vertebrates BMC Biol 2006 4 16 10.1186/1741-7007-4-16 16756667 PMC1508162

[B31] Klingenberg M Mechanism and evolution of the uncoupling protein of brown adipose tissue Trends Biochem Sci 1990 15 108 112 10.1016/0968-0004(90)90194-G 2158156

[B32] Wiersma P Chappell MA Williams JB Cold- and exercise-induced peak metabolic rates in tropical birds Proc Natl Acad Sci USA 2007 104 20866 20871 10.1073/pnas.0707683104 18093954 PMC2409233

[B33] Barre H Berne G Brebion P Cohen-Adad F Rouanet JL Loose-coupled mitochondria in chronic glucagon-treated hyperthermic ducklings Am J Physiol 1989 256 R1192 1199 2544112 10.1152/ajpregu.1989.256.6.R1192

[B34] Duchamp C Barre H Rouanet JL Lanni A Cohen-Adad F Berne G Brebion P Nonshivering thermogenesis in king penguin chicks. I. Role of skeletal muscle Am J Physiol 1991 261 R1438 1445 1661099 10.1152/ajpregu.1991.261.6.R1438

[B35] Talbot DA Duchamp C Rey B Hanuise N Rouanet JL Sibille B Brand MD Uncoupling protein and ATP/ADP carrier increase mitochondrial proton conductance after cold adaptation of king penguins J Physiol 2004 558 123 135 10.1113/jphysiol.2004.063768 15146050 PMC1664926

[B36] Dumonteil E Barre H Meissner G Expression of sarcoplasmic reticulum Ca2+ transport proteins in cold-acclimating ducklings Am J Physiol 1995 269 C955 960 7485465 10.1152/ajpcell.1995.269.4.C955

[B37] Samec S Seydoux J Dulloo AG Role of UCP homologues in skeletal muscles and brown adipose tissue: mediators of thermogenesis or regulators of lipids as fuel substrate? Faseb J 1998 12 715 724 9619450 10.1096/fasebj.12.9.715

[B38] Holmes DJ Austad SN Birds as animal models for the comparative biology of aging: a prospectus J Gerontol A Biol Sci Med Sci 1995 50 B59 66 7874580 10.1093/gerona/50a.2.b59

[B39] Gonzalez-Barroso MM Fleury C Jimenez MA Sanz JM Romero A Bouillaud F Rial E Structural and functional study of a conserved region in the uncoupling protein UCP1: the three matrix loops are involved in the control of transport J Mol Biol 1999 292 137 149 10.1006/jmbi.1999.3049 10493863

[B40] UCSC Genome Bioinformatics http://genome.ucsc.edu/

[B41] Thompson JD Gibson TJ Plewniak F Jeanmougin F Higgins DG The CLUSTAL_X windows interface: flexible strategies for multiple sequence alignment aided by quality analysis tools Nucleic Acids Res 1997 25 4876 4882 10.1093/nar/25.24.4876 9396791 PMC147148

[B42] Maddison DR Maddison WR MacClade 4: Analysis of Phylogeny and Character Evolution 2000 Sunderland, MA.: Sinauer Associates

[B43] Edgar RC MUSCLE: multiple sequence alignment with high accuracy and high throughput Nucleic Acids Res 2004 32 1792 1797 10.1093/nar/gkh340 15034147 PMC390337

[B44] Castresana J Selection of conserved blocks from multiple alignments for their use in phylogenetic analysis Mol Biol Evol 2000 17 540 552 10742046 10.1093/oxfordjournals.molbev.a026334

[B45] Guindon S Gascuel O A simple, fast, and accurate algorithm to estimate large phylogenies by maximum likelihood Syst Biol 2003 52 696 704 10.1080/10635150390235520 14530136

[B46] Guindon S Lethiec F Duroux P Gascuel O PHYML Online–a web server for fast maximum likelihood-based phylogenetic inference Nucleic Acids Res 2005 33 W557 559 10.1093/nar/gki352 15980534 PMC1160113

[B47] Posada D Crandall KA MODELTEST: testing the model of DNA substitution Bioinformatics 1998 14 817 818 10.1093/bioinformatics/14.9.817 9918953

[B48] Keane TM Creevey CJ Pentony MM Naughton TJ McLnerney JO Assessment of methods for amino acid matrix selection and their use on empirical data shows that ad hoc assumptions for choice of matrix are not justified BMC Evol Biol 2006 6 29 10.1186/1471-2148-6-29 16563161 PMC1435933

[B49] Huelsenbeck JP Ronquist F MRBAYES: Bayesian inference of phylogenetic trees Bioinformatics 2001 17 754 755 10.1093/bioinformatics/17.8.754 11524383

[B50] Page RD GeneTree: comparing gene and species phylogenies using reconciled trees Bioinformatics 1998 14 819 820 10.1093/bioinformatics/14.9.819 9918954

[B51] Tree of Life web project http://www.tolweb.org/

[B52] The NCBI Taxonomy http://www.ncbi.nlm.nih.gov/Taxonomy/

[B53] Yang Z Nielsen R Estimating synonymous and nonsynonymous substitution rates under realistic evolutionary models Mol Biol Evol 2000 17 32 43 10666704 10.1093/oxfordjournals.molbev.a026236

[B54] Yang Z PAML: a program package for phylogenetic analysis by maximum likelihood Comput Appl Biosci 1997 13 555 556 9367129 10.1093/bioinformatics/13.5.555

[B55] Pebay-Peyroula E Dahout-Gonzalez C Kahn R Trezeguet V Lauquin GJM Brandolin R Structure of mitochondrial ADP/ATP carrier in complex with carboxyatractyloside Nature 2003 426 39 44 10.1038/nature02056 14603310

