# Supplementary material for: Evolutionary history of the UCP gene family: gene duplication and selection
Source: BMC Evol Biol. 2008 Nov 3;8:306. doi: 10.1186/1471-2148-8-306 (PMC2584656; doi:10.1186/1471-2148-8-306)
Supplement: Additional file 2 — Gblocks results. Sequence alignment of UCP proteins with the selected positions underlined in blue. [file 1471-2148-8-306-S2.html]

NewNamesMuscle.pir


## Gblocks 0.91b Results

Processed file: **NewNamesMuscle.pir**  
Number of sequences: **50**  
Alignment assumed to be: **Protein**  
New number of positions: **274** (selected positions are underlined in blue)

```
                         10        20        30        40        50        60
                 =========+=========+=========+=========+=========+=========+
Zeamay19401698   ---------------------------------------------MPGDHG----SKGDI
Aratha21593775   ---------------------------------------------MADFKP-----RIEI
Cint23999        MDTEKTAVNEINCFQHKQQQQQQHKRPRTKESRVVVTILHKRNQEHIIMSPHSSTADAQL
Apime66501089    ---------------------------------------------MKQQAS----EEFP-
Anoga11676       ------------------------------------------SKMSFQRRTSEKTTELTA
MusmuUCP1        ---------------------------------------------MVNPTT----SEVQP
HosapUCP1        ---------------------------------------------MGGLTA----SDVHP
BotauUCP1        ------------------------------------------------------------
Strpur115969038  ---------------------------------------------MVGLPP----TDIKP
OanaUCP3         ---------------------------------------------MVGFKP----PDAPP
ModomUCP3        ---------------------------------------------MVGLQP----TDVPP
AnflaUCP3        ---------------------------------------------MVGLKP----TDTPP
MusmuUCP3        ---------------------------------------------MVGLQP----SEVPP
HosapUCP3        ---------------------------------------------MVGLKP----SDVPP
BotauUCP3        ---------------------------------------------MVGLQP----SERPP
Eumac13259162    ---------------------------------------------MVALKS----QEMPP
GalgalUCP3       ---------------------------------------------MVGLKP----PEVPP
Melgal16755900   ---------------------------------------------MVGLKP----PEVPP
Oana149635652    --MEAKERTPAIVIPWSLPCPTQQKLLPRGAGRDREWNSSWNHLKAKSRKP----PSAAP
SmcraUCP1        ---------------------------------------------MVGLKP----SAVPP
Modom126331519   ---------------------------------------------MVGLKP----SDVPP
TakrubUCP1       ---------------------------------------------MVGLKP----SDVPP
Tenig9630        ---------------------------------------------MVGLKP----SDVPP
CypcaUCP1        ---------------------------------------------MVGLKP----SDVPP
DareUCP3         ---------------------------------------------MVGLKP----SDVPP
DareUCP4         ---------------------------------------------MVGLKP----SDVPP
Dare50936        ---------------------------------------------MVGIKP----TDLPP
Tetnig47222581   ---------------------------------------------MVGMKA----QDVVP
TakruUCP3        ---------------------------------------------MVGMKA----PDVVP
Xetro166157878   ---------------------------------------------MVGLKP----SDVPP
Xelae147898993   ---------------------------------------------MVGLKP----SDIPP
TakrubUCP2       ---------------------------------------------MVGFGP----AEAPP
ZovivAAT99594    ---------------------------------------------MVGFGP----ADVPP
Pemar51797123    ---------------------------------------------MVGLRP----TDVPP
Lejap149930881   ---------------------------------------------MVGLRP----TDVPP
XentrUCP3        ---------------------------------------------MVGLKP----TEVPP
OanaUCP2         ---------------------------------------------MVGFKP----TDVPP
Anca1149         ---------------------------------------------MVGLKP----TEMPP
CypcaUCP2        ---------------------------------------------MVGFRA----GDVPP
DareUCP2         ---------------------------------------------MVGFRA----GDVPP
CycalbUCP2       ------------------------------------------------------------
XelaeUCP2        ---------------------------------------------MVGFKP----TDVPP
XetroUCP2        ---------------------------------------------MVGFKP----TDIPP
Anca1518         ------------------------------------------------------------
MumusUCP2        ---------------------------------------------MVGFKA----TDVPP
HosapUCP2        ---------------------------------------------MVGFKA----TDVPP
BotauUCP2        ---------------------------------------------MVGFKA----TDVPP
AnflaUCP2        ---------------------------------------------MVGFKP----TDVPP
SmmacUCP2        ------------------------------------------------------------
ModomUCP2        ---------------------------------------------MVGFKP----TEVPP
                                                              ######     ####


                         70        80        90       100       110       120
                 =========+=========+=========+=========+=========+=========+
Zeamay19401698   SFAGRFTASAIAACFAEICTIPLDTAKVRLQLQKN-------VVAAA-------------
Aratha21593775   SFLETFICSAFAACFAELCTIPLDTAKVRLQLQRK-------------------------
Cint23999        PLSLKIASAGMAGCTADLMTFPLDTVKVWLMVRGEEAKPVAANPSSSLPTKFAPVESARE
Apime66501089    -LWIKLLSAGTAACIADLATFPLDTAKVRMQIAGE-------SRPLL-------------
Anoga11676       SVPVKLLTAGSAACFADFITFPLDTAKVRLQLNPT-------------------------
MusmuUCP1        TMGVKIFSAGVSACLADIITFPLDTAKVRLQIQGE-------GQ----------------
HosapUCP1        TLGVQLFSAPIAACLADVITFPLDTAKVRLQVQGE-------CP----------------
BotauUCP1        -----IFSAGVAACVADIITFPLDTAKVRLQIQGE-------CL----------------
Strpur115969038  SVAVKLASAGLGGCIADMITFPLDTAKVRLQIQGESGSDVKKSSTQT-------------
OanaUCP3         TTTVKFFGAGTAACFADILTFPLDTAKVRLQIQGE-------------------------
ModomUCP3        TTAVKFVGAGTAACFADLLTFPLDTAKVRLQIQGE-------SQ----------------
AnflaUCP3        TTAVKVLGAGTAACFADLLTFPLDTAKVRLQIQGE-------SQ----------------
MusmuUCP3        TTVVKFLGAGTAACFADLLTFPLDTAKVRLQIQGE-------------------------
HosapUCP3        TMAVKFLGAGTAACFADLVTFPLDTAKVRLQIQGE-------NQ----------------
BotauUCP3        TTSVKFLAAGTAACFADLLTFPLDTAKVRLQIQGE-------NQ----------------
Eumac13259162    TAAIKFFSAGTAACFADLCTFPLDTAKVRLQLQGE-------VR----------------
GalgalUCP3       TAAVKFFSAGTAACIADLCTFPLDTAKVRLQIQGE-------VR----------------
Melgal16755900   TAAVKFFSAGTAACIADLCTFPLDTAKVRLQIQGE-------VR----------------
Oana149635652    TPGVKFLGAGAAACIADLVTFPLDTAKVRLQIQGE-------AQ----------------
SmcraUCP1        SPGVKFLGAGAAACIADLVTFPLDTAKVRLQIQGE-------AQ----------------
Modom126331519   TPGVKFLGAGAAACIADLVTFPLDTAKVRLQIQGE-------AQ----------------
TakrubUCP1       PLGVKMASAGAAACIADIVTFPLDTAKVRLQIQGE-------KT----------------
Tenig9630        PLGVKMASAG-AACIADIVTFPLDTAKVRLQIQGE-------KT----------------
CypcaUCP1        PLGVKVLSAGTAACIADLVTFPLDTAKVRLQIQGE-------KAVTG-------------
DareUCP3         PLTVKVLSAGTAACIADLVTFPLDTAKVRLQIQGE-------KAVTG-------------
DareUCP4         PLTVKVLSAGTAACIADLVTFPLDTAKVRLQIQGE-------KAVTG-------------
Dare50936        TAAVKFFGAGTAACFADLVTFPLDTAKVRLQIQGE-------SG----------------
Tetnig47222581   SAAVKFFGAGTAACIADLITFPLDTAKVRLQIQGE-------SQ----------------
TakruUCP3        SAAVKFFGAGTAACIADLVTFPLDTAKVRLQIQGE-------SQ----------------
Xetro166157878   TPAVKFIAAGTAACIADLFTFPLDTAKVRLQIQGE-------TT--G-------------
Xelae147898993   TPAVKFIGAGTAACIADLFTFPLDTAKVRLQIQGE-------TT--G-------------
TakrubUCP2       SAVVKFVGAGTAACIADLLTFPLDTAKVRLQIQGE-------------------------
ZovivAAT99594    SAAVKFVGAGAAGCIADLLTFPLDTAKVRLQIQGE-------LRASA-------------
Pemar51797123    TAAVKFIGAGTAACIADLITFPLDTAKVRLQVQGE-------CQR-G-------------
Lejap149930881   TAAVKFIGAGTAACIADLITFPLDTAKVRLQVQGE-------CQRGG-------------
XentrUCP3        TPLVKFVGAGTAACIADLFTFPLDTAKVRLQIQGE-------GT----------------
OanaUCP2         TATVKFLSAGTAACIADLITFPLDTAKVRLQVQGE-------SR--G-------------
Anca1149         SATVKFLSAGTAACIADLCTFPLDTAKVRLQIQGE-------SK----------------
CypcaUCP2        TATVKFIGAGTAACIADLFTFPLDTAKVRLQIQGE-------SK--I-------------
DareUCP2         TATVKFIGAGTAACIADLFTFPLDTAKVRLQIQGE-------NK--A-------------
CycalbUCP2       ------------------------------------------------------------
XelaeUCP2        TAAVKFIGAGTAACIADLFTFPLDTAKVRLQIQGE-------SK----------------
XetroUCP2        TAAVKFVGAGTAACIADLFTFPLDTAKVRLQIQGE-------NK----------------
Anca1518         ------------------------------------------------------------
MumusUCP2        TATVKFLGAGTAACIADLITFPLDTAKVRLQIQGE-------SQ--G-------------
HosapUCP2        TATVKFLGAGTAACIADLITFPLDTAKVRLQIQGE-------SQ--G-------------
BotauUCP2        TATVKFLGAGTAACIADLITFPLDTAKVRLQIQGE-------RQ--G-------------
AnflaUCP2        TATVKFLGAGTAACIADLITFPLDTAKVRLQIQGE-------SQ--G-------------
SmmacUCP2        -------------------------AKVRLQIQGE-------SQ--G-------------
ModomUCP2        TATVKFLGAGTAACIADLITFPLDTAKVRLQIQGE-------SQ--G-------------
                 ###################################                         


                        130       140       150       160       170       180
                 =========+=========+=========+=========+=========+=========+
Zeamay19401698   -------------------------------A-SGDAAPALPKY----------------
Aratha21593775   --------------------------------IPTGDGENLPKY----------------
Cint23999        SSTGIYKRQAVASSQKLDKPGLKKFFRPTTMTTSLQKKTTAIRFSAYNAKLSKIVPSPIK
Apime66501089    ---------------------------------LATTDGSMLAM----------------
Anoga11676       ---------------------------------SVPATQH-VQY----------------
MusmuUCP1        -------------------------------------ASSTIRY----------------
HosapUCP1        -------------------------------------TSSVIRY----------------
BotauUCP1        ---------------------------------ISSA----IRY----------------
Strpur115969038  ---------------------------------TGKELRASFRY----------------
OanaUCP3         -----------------------------------PGAGQPVRY----------------
ModomUCP3        ---------------------------------SEKAIQN-VRY----------------
AnflaUCP3        ---------------------------------AEQAIQN-VRY----------------
MusmuUCP3        -----------------------------------NPGAQSVQY----------------
HosapUCP3        ---------------------------------AVQTARL-VQY----------------
BotauUCP3        ---------------------------------AALAARS-AQY----------------
Eumac13259162    ---------------------------------IPRVSGA-VEY----------------
GalgalUCP3       ---------------------------------IPRSTNT-VEY----------------
Melgal16755900   ---------------------------------IPRSTNT-VEY----------------
Oana149635652    ---------------------------------VASA----IRY----------------
SmcraUCP1        ---------------------------------SAGA----VRY----------------
Modom126331519   ---------------------------------TMDA----VRY----------------
TakrubUCP1       ---------------------------------AVEG----IRY----------------
Tenig9630        ---------------------------------AVEG----IRY----------------
CypcaUCP1        ---------------------------------AAKG----IRY----------------
DareUCP3         ---------------------------------AAKG----IRY----------------
DareUCP4         ---------------------------------AAKG----IRY----------------
Dare50936        ---------------------------------TAPGSAV-LKY----------------
Tetnig47222581   ---------------------------------KVGEGCG-AKY----------------
TakruUCP3        ---------------------------------IVEGSRA-TKY----------------
Xetro166157878   ---------------------------------SGAANG--IRY----------------
Xelae147898993   ---------------------------------SAAVNG--IRY----------------
TakrubUCP2       ---------------------------------GKGAGASAVKY----------------
ZovivAAT99594    ---------------------------------AAGKGSA-VRY----------------
Pemar51797123    ---------------------------------EGAARSAGVQY----------------
Lejap149930881   ---------------------------------EGAARSAGVQY----------------
XentrUCP3        ---------------------------------SVKDTKV-LRY----------------
OanaUCP2         ---------------------------------PSRVPAG-PQY----------------
Anca1149         ---------------------------------SSRAAKD-VRY----------------
CypcaUCP2        ---------------------------------PVNTGHGPVKY----------------
DareUCP2         ---------------------------------STNMGRGPVKY----------------
CycalbUCP2       ------------------------------------------------------------
XelaeUCP2        ---------------------------------AVHMKT--ASY----------------
XetroUCP2        ---------------------------------VVNVKA--AQY----------------
Anca1518         ------------------------------------------------------------
MumusUCP2        ---------------------------------LVRTAAS-AQY----------------
HosapUCP2        ---------------------------------PVRATAS-AQY----------------
BotauUCP2        ---------------------------------PMQAAAS-AQY----------------
AnflaUCP2        ---------------------------------AIRASSTTAQY----------------
SmmacUCP2        ---------------------------------AIRASTT-AQY----------------
ModomUCP2        ---------------------------------AIRTSSTGAQY----------------
                                                                             


                        190       200       210       220       230       240
                 =========+=========+=========+=========+=========+=========+
Zeamay19401698   ---------------------R----GLLGTAATIAREEGA----------AALWKGIVP
Aratha21593775   ---------------------R----GSIGTLATIAREEGI----------SGLWKGVIA
Cint23999        PYGAARTNFGVAAVAQSQSTASRSSAGLVRTVINGVKQNGF----------LSLYGGFAA
Apime66501089    ---------------------RNTQPGLWRTVKNIVRLEGARAVSLSEGGYRSLYGGLSA
Anoga11676       ---------------------R----GLVGTITTITRQEGF----------RTLYNGLSA
MusmuUCP1        ---------------------K----GVLGTITTLAKTEGL----------PKLYSGLPA
HosapUCP1        ---------------------K----GVLGTITAVVKTEGR----------MKLYSGLPA
BotauUCP1        ---------------------K----GVLGTIITLAKTEGP----------VKLYSGLPA
Strpur115969038  ---------------------R----GVFGTIWTIIRQEGP----------RGLYNGLIP
OanaUCP3         ---------------------R----GVLGTILTMARTEGP----------GSLYGGLVA
ModomUCP3        ---------------------R----GVLGTITTMVKTEGP----------SSLYNGLVA
AnflaUCP3        ---------------------R----GVLGTLLTMAKTEGP----------ASLYNGLVA
MusmuUCP3        ---------------------R----GVLGTILTMVRTEGP----------RSPYSGLVA
HosapUCP3        ---------------------R----GVLGTILTMVRTEGP----------CSPYNGLVA
BotauUCP3        ---------------------R----GVLGTILTMVRTEGP----------RSLYSGLVA
Eumac13259162    ---------------------R----GVLGTLSTMVRTEGA----------RSLYRGLAA
GalgalUCP3       ---------------------R----GVLGTLSTMVRTEGP----------RSLYSGLVA
Melgal16755900   ---------------------R----GVLGTLSTMVRTEGP----------RSLYSGLVA
Oana149635652    ---------------------K----GVLGTIVTLVKTEGP----------RSLYSGLIA
SmcraUCP1        ---------------------K----GVLGTIVTLVKTEGP----------RSLYSGLHA
Modom126331519   ---------------------K----GILGTIITLVKTEGP----------RSLYNGLHA
TakrubUCP1       ---------------------R----GVFGTISTMVRTEGP----------RSLYNGLVA
Tenig9630        ---------------------R----GVFGTISTMIRTEGP----------RSLYNGLVA
CypcaUCP1        ---------------------R----GVFGXISTMVRTEGP----------RSLYNGLVA
DareUCP3         ---------------------K----GVFGTISTMMRTEGP----------RSLYNGLVA
DareUCP4         ---------------------K----GVFGTISTMMRTEGP----------RSLYNGLVA
Dare50936        ---------------------R----GVFGTITTMVRTEGA----------RSLYNGLVA
Tetnig47222581   ---------------------R----GVFGTITTMVRTEGP----------RSLYSGLVA
TakruUCP3        ---------------------R----GVFGTITTMVRTEGP----------RSLYSGLVA
Xetro166157878   ---------------------K----GVFGTISTIVKTEGP----------KSLYNGLVA
Xelae147898993   ---------------------K----GVFGTLSTIVKTEGP----------KSLYNGLVA
TakrubUCP2       ---------------------R----GMFGTITTMVRTEGP----------RSLYSGLVA
ZovivAAT99594    ---------------------R----GVFGTITTMVRTEGP----------RSLYSGLVA
Pemar51797123    ---------------------R----GVFGTIAAMVRTEGP----------RSLYSGLVA
Lejap149930881   ---------------------R----GVFGTIAAMVRTEGP----------RSLYSGLVA
XentrUCP3        ---------------------K----GVFGTIKTMVKTEGA----------TSLYNGLVA
OanaUCP2         ---------------------R----GVLGTILTVARTEGP----------GSLYSGLVA
Anca1149         ---------------------K----GVFGTITTMVKMEGP----------RSLYNGLVA
CypcaUCP2        ---------------------R----GVFGTISTMVRVEGP----------RSLYSGLVA
DareUCP2         ---------------------R----GVFGTISTMVRVEGP----------RSLYSGLVA
CycalbUCP2       -----------------------------------------------------------A
XelaeUCP2        ---------------------K----GVFGTISTMVKMEGP----------KSLYNGLAA
XetroUCP2        ---------------------K----GVFGTISTMVKTEGP----------KSLYNGLVA
Anca1518         ------------------------------------------------------------
MumusUCP2        ---------------------R----GVLGTILTMVRTEGP----------RSLYNGLVA
HosapUCP2        ---------------------R----GVMGTILTMVRTEGP----------RSLYNGLVA
BotauUCP2        ---------------------R----GVLGTILTMVRTEGP----------RSLYSGLVA
AnflaUCP2        ---------------------R----GVMGTILTMVKTEGP----------GSLYNGLVA
SmmacUCP2        ---------------------R----GVLGTILTMVKTEGP----------GSLYSGLVA
ModomUCP2        ---------------------R----GVMGTILTMVKTEGP----------GSLYNGLVA
                                           ###############          #########


                        250       260       270       280       290       300
                 =========+=========+=========+=========+=========+=========+
Zeamay19401698   GLHRQCIYGGLRIGLYEPVKSFYV-----GKDH--VGDVPLSKKIAAGFTTGAIAISIAN
Aratha21593775   GLHRQCIYGGLRIGLYEPVKTLLV-----GSDF--IGDIPLYQKILAALLTGAIAIIVAN
Cint23999        GLQRQVSFCAVRIGLYDSVKGFYMQLIPTSTNS--KQ---VPQRILAGATTAIMAATMFQ
Apime66501089    GLQRQMCFASIRLGLYDGVKSRYA-GIIDGNNRSASGSKSISVRIAAGITTGALAVLFAQ
Anoga11676       GLQRQLCFCSIRLGLYDTVKTFY------GSLL--KAGLQIGTRVLAGLTTGGAAVMIAQ
MusmuUCP1        GIQRQISFASLRIGLYDSVQEYFS----SGRETP-AS---LGNKISAGLMTGGVAVFIGQ
HosapUCP1        GLQRQISSASLRIGLYDTVQEFLT----AGKETA-PS---LGSKILAGLTTGGVAVFIGQ
BotauUCP1        GLQRQISLASLRIGLYDTVQEFFT----TGKE---AS---LGSKISAGLMTGGVAVFIGQ
Strpur115969038  GLQRQMCFASVRIGLYDSVKGFYA---EYGG----VN---IFTRISAGITTGACAVLTAQ
OanaUCP3         GLQRQMSFASVRIGLYDSVKQLYT---PAGSEQ--SS---IAVRLLAGCTTGAMAVTCAQ
ModomUCP3        GLHRQMSFASIRIGLYDSVKQFYT---PKGAEN--SS---IIVRILAGCTTGAMAVTCAQ
AnflaUCP3        GLQRQMSFASIRIGLYDSVKQFYT---PKGAEN--SS---IMIRILAGCTTGAMAVSCAQ
MusmuUCP3        GLHRQMSFASIRIGLYDSVKQFYT---PKGADH--SS---VAIRILAGCTTGAMAVTCAQ
HosapUCP3        GLQRQMSFASIRIGLYDSVKQVYT---PKGADN--SS---LTTRILAGCTTGAMAVTCAQ
BotauUCP3        GLQRQMSFASIRIGLYDSVKQFYT---PKGSDH--SS---IITRILAGCTTGAMAVTCAQ
Eumac13259162    GLQRQMSFASIRIGLYDSVKQLYT---PKGAES--TG---LAPRLLAGCTTGAVAVACAQ
GalgalUCP3       GLQRQMSFASIRIGLYDSVKQLYT---PKGAES--TG---LLARLLAGCTTGAVAVTCAQ
Melgal16755900   GLQRQMSFASIRIGLYDSVKQLYT---PKGAES--TG---LLARLLAGCTTGAVAVTCAQ
Oana149635652    GLQRQMSFASIRIGLYDTAKQFYT----NGKET--AG---IGSRILAGCTTGGMAVVIAQ
SmcraUCP1        GLQRQMSFASIRIGLYDTAKQFYN----NGRET--AG---IGSRILAGCTTGGLAVIVAQ
Modom126331519   GLQRQISFASIRIGLYDTAKQLYN----NGRET--AG---IGSRILAGCTTGGLAVIVAQ
TakrubUCP1       GLQRQLCFASVRIGLYDSVRDFYT----GGKEN--PN---VLIRILAGCTTGAMAVSFAQ
Tenig9630        GLQRQLCFASIRIGLYDNVKNFYT----GGKDN--PS---VLIRILAGCTTGAMAVSFAQ
CypcaUCP1        GLQRQMAFASIRIGLYDNVKSFYT----RGKDN--PN---VGIRILAGCTTGALAVSVAQ
DareUCP3         GLQRQMAFASIRIGLYDNVKSFYT----RGKDN--PN---VAVRILAGCTTGAMAVSMAQ
DareUCP4         GLQRQMAFASIRIGLYDNVKSFYT----RGKDN--PN---VAVRILAGCTTGAMAVSMAQ
Dare50936        GLQRQMSFASVRIGLYDSMKQFYT----RGSEN--AS---IVTRLLAGCTTGAMAVAFAQ
Tetnig47222581   GLQRQMSFASVRIGLYDSMKQFYT----RGTES--AG---IVTRLMAGCTTGAMAVAFAQ
TakruUCP3        GLQRQMSFASVRIGLYDSMKQFYT----RGTDS--AG---IVTRLMAGCTTGAMAVAFAQ
Xetro166157878   GLQRQMSFASIRIGLYDTVKLFYT----NGKEK--AG---IGSRILAGCTTGALAVTVAQ
Xelae147898993   GLQRQMSFASIRIGLYDTVKLFYT----NGKEK--AG---IGSRILAGCTTGALAVTVAQ
TakrubUCP2       GLQRQMSFASVRIGLYDSVKQFYT----RGSDC--IG---VGTRLLAGCTTGAMAVALAQ
ZovivAAT99594    GLQRQMSFASVRIGLYDSVKQFYT----KGSDH--VG---IGIRLLAGCTTGAMAVAFAQ
Pemar51797123    GLQRQMSFASVRIGLYDSVKNFYT----NGAEH--AG---IGCRLLAGCTTGAMAVTFAQ
Lejap149930881   GLQRQMSFASVRIGLYDSVKNFYT----NGAEH--AG---IGCRLLAGCTTGAMAVTFAQ
XentrUCP3        GLQRQMSFASIRIGLYDSVKQFYC----RQSES--SG---VACRLLAGCTTGAMAVTLAQ
OanaUCP2         GLQRQMSFASVRIGLYDSVKQFYT---XXXXXX--AD---FESRYIVGCTTGALAVGLAQ
Anca1149         GLQRQMSFASIRIGLYDSVKQFYT-------------------RLLAGCTTGAMAVTCAQ
CypcaUCP2        GLQRQMSFASVRIGLYDSVKQFYT----KGSEH--VG---IGSRLMAGCTTGAMAVALAQ
DareUCP2         GLQRQMSFASVRIGLYDSVKQFYT----KGSDH--AG---IGSRLMAGCTTGAMAVAVAQ
CycalbUCP2       GLQSQMSFASVRIGLYDSVKQFYT----KGSEH--VG---IGSRLLAGCTTGAMAVAIAQ
XelaeUCP2        GLQRQMSFASVRIGLYDSVKQFYT----KGSEH--AG---IGSRLAAGCTTGAMAVAVAQ
XetroUCP2        GLQRQMSFASVRIGLYDSVKQFYT----KGSEH--VG---IGSRLAAGCTTGAMAVAVAQ
Anca1518         -----MSFASVRIGLYDSVKQFYT----KGSE----G---IGSRLLAGCTTGAMAVAVAQ
MumusUCP2        GLQRQMSFASVRIGLYDSVKQFYT----KGSEH--AG---IGSRLLAGSTTGALAVAVAQ
HosapUCP2        GLQRQMSFASVRIGLYDSVKQFYT----KGSEH--AS---IGSRLLAGSTTGALAVAVAQ
BotauUCP2        GLQRQMSFASVRIGLYDSVKQFYT----KGSEH--AG---IGSRLLAGSTTGALAVAVAQ
AnflaUCP2        GLQRQMSFASVRIGLYDSVKQFYT----KGAEH--AS---IGSRLLAGCTTGALAVAVAQ
SmmacUCP2        GLQRQMSFASVRIGLYDSVKQFYT----KGSEH--AS---IGSRLLAGCTTGALAVAVAQ
ModomUCP2        GLQRQMSFASVRIGLYDSVKQFYT----KGSEH--AG---IGSRLLAGCTTGALAVGVAQ
                 ########################     ###        ####################


                        310       320       330       340       350       360
                 =========+=========+=========+=========+=========+=========+
Zeamay19401698   PTDLVKVRLQAEGKLAPGV--PRRYTGAMDA---YSKIARQEGVAALWTGLGPNVARNAI
Aratha21593775   PTDLVKVRLQSEGKLPAGV--PRRYAGAVDA---YFTIVKLEGVSALWTGLGPNIARNAI
Cint23999        PTEVVKIRMQAQTRLP-AS--QRTYTSSVQA---YRSIFRHGGIPELWKGLGANATRLSV
Apime66501089    PTDVVKVRLQAGSN---GR--SVRYSSTLQA---YKNIAAEEGTRGLWKGTVPNISRNAI
Anoga11676       PTDVVKVRFQAATRS--ST--GRRYASTLEA---YRTIHREEGVRGLWRGAMPNVGRNAI
MusmuUCP1        PTEVVKVRMQAQSHLH-GI--KPRYTGTYNA---YRVIATTESLSTLWKGTTPNLMRNVI
HosapUCP1        PTEVVKVRLQAQSHLH-GI--KPRYTGTYNA---YRIIATTEGLTGLWKGTTPNLMRSVI
BotauUCP1        PTEVVKVRLQAQSHLH-GP--KPRYTGTYNA---YRIIATTEGLTGLWKGTSPNLTTNVI
Strpur115969038  PTDVVKIRLQAQGNAVLNG-APKRYTGALNA---YQTIAKEEGVRGLWKGTMPNIVRNSV
OanaUCP3         PTDVVKVRFQACVQLE-PG--SRKYSGTVDA---YRTIAREEGVRGLWKGTVPNITRNAI
ModomUCP3        PTDVVKVRFQASVRLGPGS--CRKYSGTMDA---YRTIAREEGIRGLWKGTLPNITRNAI
AnflaUCP3        PTDVVKVRFQASVRMGPGT--SRKYNGTMDA---YRTIAREEGIRGLWKGTLPNITRNAI
MusmuUCP3        PTDVVKVRFQAMIRLGTGG--ERKYRGTMDA---YRTIAREEGVRGLWKGTWPNITRNAI
HosapUCP3        PTDVVKVRFQASIHLG-PSRSDRKYSGTMDA---YRTIAREEGVRGLWKGTLPNIMRNAI
BotauUCP3        PTDVVKIRFQASMHTGLGG--NRKYSGTMDA---YRTIAREEGVRGLWKGILPNITRNAI
Eumac13259162    PTDVVKVRFQAHGAMP-ES--TRRYNGTLDA---YRTIAREEGVRGLWRGTLPNIARNAV
GalgalUCP3       PTDVVKVRFQALGALP-ES--NRRYSGTVDA---YRTIAREEGVRGLWRGTLPNIARNSI
Melgal16755900   PTDVVKVRFQALGALP-ES--NRRYSGTVDA---YRTIAREEGVRGLWRGTLPNIARNAI
Oana149635652    PTDVVKVRFQAQSNLH-GA--KPRYSGTLQA---YKSIAAEEGVRGLWKGTLPNVTRNAI
SmcraUCP1        PTDVVKVRLQAQSNLS-GA--KPRYTGTFHA---YKTIATEEGARGLWKGTTPNVTRNAI
Modom126331519   PTDVVKVRLQAQSSLS-GA--KPRYTGTFHA---YKKIASEEGTRGLWKGTMPNVARNAI
TakrubUCP1       PTDVVKVRFQAQMNLN-SV--ARRYSGTMQA---YKHIYQNEGFRGLWKGTLPNITRNAL
Tenig9630        PTDVVKVRFQAQMNLN-SV--ARRYSG-MQA---YKHIYQNEGFRGLWKGTLPNITRNAL
CypcaUCP1        PTDVVKVRFQAQMNLQ-GV--GRRYSGTMQA---YRQIFQHEGLRGLWKGTLPNITRNAL
DareUCP3         PTDVVKVRFQAQMNLQ-GV--GRRYNGTMQA---YRQIFQLEGLRGLWKGTLPNITRNAL
DareUCP4         PTDVVKVRFQAQMNLQ-GV--GRRYNGTMQA---YRQIFQLEGLRGLWKGTLPNITRNAL
Dare50936        PTDVVKVRFQAQVRHT-DG--GKRYNGTMDA---YRTIARDEGVRGLWKGCMPNITRNAI
Tetnig47222581   PTDVVKVRFQAQVRVA-DG--GRRYNGTLDA---YKTIARDEGVRGLWKGCLPNITRNAI
TakruUCP3        PTDVVKVRFQAQVREA-ES--GRRYNGTLDA---YKTIARDEGVRGLWKGCLPNITRNAI
Xetro166157878   PTDVVKVRFQAQANLQ-GV--KRRYNGTMDA---YKTIAKKEGVRGLWKGTFPNVTRNAI
Xelae147898993   PTDVVKVRFQAQANLH-GV--KKRYNGTMDA---YKTIAKKEGIKGLWKGTFPNVTRNAI
TakrubUCP2       PTDVVKVRFQAQARSP-GE--SRRYCSTIDA---YKTIAKEEGVHGLWKGTAPNIARNAI
ZovivAAT99594    PTDVVKVRLQAQARRP-GQ--ARRYCSTIDA---YKTIAKEEGIRGLWKGTAPNIARNAI
Pemar51797123    PTDVVKVRFQAQVNML-GT--SKRYSGNHERLQDHRTGRRASVASG--KAPAPNITRNAI
Lejap149930881   PTDVVKVRFQAQVNML-GT--SKRYSGTINA---YKTIAREEGVRGLWKGTGPNITRNAI
XentrUCP3        PTDVVKVRFQAHIKVM-DG--ERRYNGTVDA---YKTIAKEEGLRGLWKGTIANITRNAI
OanaUCP2         PTDVVKVRFQAQARA--AG--SRRYQGTVDA---YKTIAREEGIRGLWKGTSPNVARNAI
Anca1149         PTDVVKVRFQA-------------YNGTVDA---YRTIAREEGVRGLWKGKL--------
CypcaUCP2        PTDVVKVRFQAQNSA--GA--NKRYHGTMDA---YRTIAKEEGFRGLWKGTGPNITRNAI
DareUCP2         PTDVLKVRFQAQVSA--GA--SKRYHSTMDA---YRTIAKEEGFRGLWKGTGPNITRNAI
CycalbUCP2       PTDVVKVRFQAQANV--SS--ARRYKGTMDA---YKTIARQEGVRGLWKGTAPNITRNAL
XelaeUCP2        PTDVVKVRFQAQANS--SA--NRRYKGTMDA---YRTIAREEGMRGLWKGTVPNITRNAI
XetroUCP2        PTDVVKVRFQAQANS--SA--NRRYKGTMHA---YRTIAREEGMRGLWKGTAPNITRNAI
Anca1518         PTDVVKVRFQAQARM--EG--SKRYQGTLDA---YKTIAREEGIRGLWKGTSPNITRNAL
MumusUCP2        PTDVVKVRFQAQARA--GG--GRRYQSTVEA---YKTIAREEGIRGLWKGTSPNVARNAI
HosapUCP2        PTDVVKVRFQAQARA--GG--GRRYQSTVNA---YKTIAREEGFRGLWKGTSPNVARNAI
BotauUCP2        PTDVVKVRFQAQARA--GA--GRRYQSTVEA---YKTIAREEGFRGLWKGTSPNVARNAI
AnflaUCP2        PTDVVKVRFQAQARG--GG--SRRYQGTVDA---YKTIAREEGLRGLWRGTSPNIARNAI
SmmacUCP2        PTDVVKVRFQAQAQAR-GS--SRRYQGTMDA---YKTIAREEGLRGLWKGTLPNVARNAI
ModomUCP2        PTDVVKVRFQAQARA--GG--SRRYQGTMDA---YKTIAREEGLRGLWKGTSPNVARNAI
                 ############          #########   ##########################


                        370       380       390       400       410       420
                 =========+=========+=========+=========+=========+=========+
Zeamay19401698   INAAELASYDQVKQSILKLPGFKDDVVTHLFAGLGAGFFAVCVGSPVDVVKSRMMGDSA-
Aratha21593775   VNAAELASYDQIKETIMKIPFFRDSVLTHLLAGLAAGFFAVCIGSPIDVVKSRMMGDST-
Cint23999        VNVSELVTYDLVKEFILDHKILNDNPICHFTSAFISGFVTTLVASPVDVVKTRYMNSPLG
Apime66501089    VNVAEIVCYDIIKDFILEHGYLRDGIPCHITAAVAAGLCTTLAASPVDVVKTRYMNSAPG
Anoga11676       VNVAEIVCYDVVKDCLLLYAHMPNDIRCHFSAAVVAGLAATIVASPVDVVKTRYMNSPRG
MusmuUCP1        INCTELVTYDLMKGALVNNKILADDVPCHLLSALVAGFCTTLLASPVDVVKTRFINSLPG
HosapUCP1        INCTELVTYDLMKEAFVKNNILADDVPCHLVSALIAGFCATAMSSPVDVVKTRFINSPPG
BotauUCP1        INCTELVTYDLMKEALVKNKLLADDVPCHFVSAVVAGFCTTVLSSPVDVVKTRFVNSSPG
Strpur115969038  VNASEVVAYDLIKEAILKRRYLKDEFPCHFIAAFGAGFVTTCVATPVDVVKTRFMNSSPG
OanaUCP3         VNCAEMVTYDLIKESLTDHHLMTDDFPCHFVSAFGAGFCATVVASPVDVVKTRYMNSAPG
ModomUCP3        VNCAEMVTYDMIKEALIDRHLMTDNFPCHFISAFSAGFCATVVASPVDVVKTRYINSPPG
AnflaUCP3        VNCAEMVTYDMIKEALIDHHLMTDNFPCHFVSAFAAGFCATVVANPVDVVKTRYINAPPG
MusmuUCP3        VNCAEMVTYDIIKEKLLESHLFTDNFPCHFVSAFGAGFCATVVASPVDVVKTRYMNAPLG
HosapUCP3        VNCAEVVTYDILKEKLLDYHLLTDNFPCHFVSAFGAGFCATVVASPVDVVKTRYMNSPPG
BotauUCP3        VNCGEMVTYDIIKEKLLDYHLLTDNFPCHFVSAFGAGFCATLVASPVDVVKTRYMNSPPG
Eumac13259162    INCGELVTYDLIKDALLREHLMADDVPCHFVAAFGAGFCATVVASPVDVVKTRYMNAGPG
GalgalUCP3       INCGELVTYDLIKDTLLRAQLMTDNVPCHFVAAFGAGFCATVVASPVDVVKTRYMNASPG
Melgal16755900   INCGELVTYDLIKDTLLRAQLMTDNVPCHFVAAFGAGFCATVVASPVDVVKTRYMNASPG
Oana149635652    VNCTELVTYDIIKETILKHNLLTDNLPCHFLSASGAGFCTTVVASPVDVVKTRYMNSPPG
SmcraUCP1        VNSAELVTYDLIKENLLKYNILTDNLPCHFVSAFGAGFCTTVVASPVDVVKTRYMNSPPG
Modom126331519   VNSAELVTYDLIKENLLKYNLLTDNLPCHFVSAFGAGFCTTVVASPVDVVKTRYMNSPPG
TakrubUCP1       VNCTELVTYDMIKEAILRHKLMSDNLPCHFVSAFGAGFVTTVIASPVDVVKTRYMNSPPG
Tenig9630        VNCTELVTYDMIKEAILRHKLMSDNLPCHFVSAFGAGFVTTVIASPVDVVKTRYMNSPPG
CypcaUCP1        VNCTELVSYDLIKEALLKHKLMSDNLPCHFVSAFGAGFVTTVIASPVDVVKTRYMNSPPE
DareUCP3         VNCTELVSYDLIKEAILKHRLLSDNLPCHFVSAFGAGFITTVIASPVDVVKTRYMNSPPG
DareUCP4         VNCTELVSYDLIKEAILKHRLLSDNLPCHFVSAFGAGFITTVIASPVDVVKTRYMNSPPG
Dare50936        VNCAELVTYDIIKDLILKYDLMTDNLPCHFTAAFGAGFCTTIVASPVDVVKTRFMNSSAG
Tetnig47222581   VNCAELVTYDLIKELILKYGLMTDDLPCHFTAAFGAGFCTTVVASPVDVVKTRFMNSGSG
TakruUCP3        VNCAELVTYDLIKELILKYDLMTDNLPCHFTAAFGAGFCTTVVASPVDVVKTRFMNSTSG
Xetro166157878   VNCTELVTYDVIKENLLHYKLMTDNLPCHFVSAFGAGFCTTVIASPVDVVKTRYMNSPPG
Xelae147898993   VNCTELVTYDLIKENLLHHKLMTDNLPCHFVSAFGAGFCTTVIASPVDVVKTRYMNSPPG
TakrubUCP2       VNCTELVTYDLIKDTLLKSTPLTDNLPCHFVSAFGAGLCTTVIASPVDVVKTRYMNSSPG
ZovivAAT99594    VNCTELVTYDFIKDSLLKSTPLTDNLPCHFVSAFGAGLCTTVTASPVDVVKTRYMNAALG
Pemar51797123    VNCAELVTYDIIKDTILKYKLLN-------------------------------------
Lejap149930881   VNCAELVTYDIIKDTILKYKLLTDNLPCHFVSAFGAGFCTTVVASPVDVVKTRYMNSAPG
XentrUCP3        VNCAELVTYDLIKETILNQRLMTDNLPCHFVAAFGAGFCATVVASPVDVVKTRYMNSPAG
OanaUCP2         VNCAELVTYDLIKDALLRGGLMADDLPCHLTSAFGAGFCTTVIASPVDVVKTRYMNSASG
Anca1149         -----------------------DNFPCHFVAAFGAGFCATVVASPVDVVKTRYMNSIPG
CypcaUCP2        VNCTELVTYDLIKDALLKSSLMTDDLPCHFTSAFGAGFCTTVIASPVDVVKTRYMNSAPG
DareUCP2         VNCTELVTYDLIKDALLKSSLMTDDLPCHFTSAFGAGFCTTIIASPVDVVKTRYMNSAQG
CycalbUCP2       VNCTELVTYDLIKDALLKSNLMSDTLPCHFTSAFGAGFCTTVIASPVDVVKTRYMN----
XelaeUCP2        VNCTELVTYDLIKDSILKANIMTDNLPCHFTSAFGAGFCTTVIASPVDVVKTRYMNSAKG
XetroUCP2        VNCTELVTYDIIKDSLLKANIMTDNLPCHFTSAFGAGFCTTVIASPVDVVKTRYMNSAKG
Anca1518         VNCAELVTYDLIKDMILRYNLMSDNLPCHFTSAFGAGFCTTVIASPVDVVKTRYMN----
MumusUCP2        VNCAELVTYDLIKDTLLKANLMTDDLPCHFTSAFGAGFCTTVIASPVDVVKTRYMNSALG
HosapUCP2        VNCAELVTYDLIKDALLKANLMTDDLPCHFTSAFGAGFCTTVIASPVDVVKTRYMNSALG
BotauUCP2        VNCAELVTYDLIKDTLLKAHLMTDDLPCHFTSAFGAGFCTTVIASPVDVVKTRYMNSALG
AnflaUCP2        VNCAELVTYDLIKDALLKAHLMTDDLPCHFISAFGAGFCTTIIASPVDVVKTRYMNSAAG
SmmacUCP2        VNCAELVTYDLIKDALLKAYLMTDDLPCHFTSAFGAGFCTTIIASPVDVVKTRYMNSATG
ModomUCP2        VNCAELVTYDLIKDALLKAHLMTDDLPCHFTSAFGAGFCTTIIASPVDVVKTRYMNSASG
                 ############################################################


                        430       440       450       460       470       480
                 =========+=========+=========+=========+=========+=========+
Zeamay19401698   -YKSTLDCFVKTLKNDGPLAFYKGFLPNFARLGSWNVIMFLTLEQVQKLFVRKATS----
Aratha21593775   -YRNTVDCFIKTMKTEGIMAFYKGFLPNFTRLGTWNAIMFLTLEQVKKVFLREVLYD---
Cint23999        TYKNPIHCTKTLFMQEGMKAFYKGFVPSYLRLGTWNIVMFVSYEEYKVLAHAYNNQHRVK
Apime66501089    EYKGVKDCAVRMMMKEGPSAFYKGFVPSFTRLVSWNIVLWITYEQFKVYAKKLNQ-----
Anoga11676       QYRGAIDCAIRMGAKEGVAAFYKGFVPSFARLVSWNVVMWISYEQLKLVIFNRNQS----
MusmuUCP1        QYPSVPSCAMSMYTKEGPTAFFKGFVASFLRLGSWNVIMFVCFEQLKKELMKSRQTVDCT
HosapUCP1        QYKSVPNCAMKVFTNEGPTAFFKGLVPSFLRLGSWNVIMFVCFEQLKRELSKSRQTMDCA
BotauUCP1        QNTSVPNCAMMMLTREGPSAFFKGFVPSFLRLGSWNI-MFVCFERLKQELMKCRHTMDCA
Strpur115969038  QYRGATECATQMFQKEGLLAFYKGFTPQFLRLGSWNIVMFVCYEQLKRAMILSTQHN---
OanaUCP3         QYPGVFGC-MKAVAGEGPAAFYKGFTPSFLRLGSWNVVMFVTYEQLKRALMEVRVSWESP
ModomUCP3        RYSSTVDCMLKTLSQEGPTAFYKGFTPSFLRLGSWNVMMFVTYEQLKRALMKLQMSWESS
AnflaUCP3        RYGSTLDCMLKTLRLEGPTAFYKGFTPSFLRLGSWNVMMFVTYEQLKRALMKLQMSWESS
MusmuUCP3        RYRSPLHCMLKMVAQEGPTAFYKGFVPSFLRLGAWNVMMFVTYEQLKRALMKVQVLRESP
HosapUCP3        QYFSPLDCMIKMVAQEGPTAFYKGFTPSFLRLGSWNVVMFVTYEQLKRALMKVQMLRESP
BotauUCP3        QYHSPFDCMLKMVTQEGPTAFYKGFTPSFLRLGSWNVVMFVTYEQMKRALMKVQMLRDSP
Eumac13259162    QYRNALSCLLALLMQDGITGFYKGFVPSFLRLGSWNVVMFICYEQLQRAAVLALS-----
GalgalUCP3       QYRNVPSCLLALLLQDGIAGLYKGFVPSFLRLGSWNVVMFISYEQLQRVVMLARS---AP
Melgal16755900   QYRNVPSCLLALLMQDGISGLYKGFVPSFLRLGSWNVVMFISYEQLQRVVMLARS---AP
Oana149635652    QYLSALNCAWTMLTREGPTAFYKGCVPSFLRLGSWNIVMFVSYEQLKRAMMKARPTIDCA
SmcraUCP1        QYTSAPKCAWTMLTREGPTAFYKGFVPSFLRLGSWNVVMFVSYEQLKRAMMRSGPTIDCA
Modom126331519   QYTSAPKCAWTMLWREGLTAFYKGFVPSFLRLGSWNVIMFVSYEQLKRALMKSKPTIDCT
TakrubUCP1       QYRSAINCAWTMMTKEGPTAFYKGFVPSFLRLGSWNIVMFVSFEQIKRAMMVTKKKIEAK
Tenig9630        QYRSAINCAWTMMTKEGPTAFYKGFVPSFLRLGSWNIVMFVSFEQIKRAMMVTKKKIEAK
CypcaUCP1        QYRSSLNCAWTMMTKEGPTAFYKGFVPSFLRLGSWNVVMFVSFEQLKRAMMMSRSRIEAT
DareUCP3         QYSGSTNCAWTMLTKEGPTAFYKGFVPSFLRLGSWNVVMFVSFEQLKRAMMVSRNRIEAA
DareUCP4         QYSSSTNCAWTMLTKEGPTAFYKGFVPSFLRLGSWNVVMFVSFEQLKRAMMVSRNRIEAA
Dare50936        QYGSALNCALMMLTKEGPAAFYKGFMPSFLRLGSWNIVMFVSYEQIKRCMTRMQHSWESP
Tetnig47222581   QYSSAVNCALTMLRQEGPTAFYKGFMPSFLRLGSWNIVMFVSYEQIKRGMCRTQQYWESP
TakruUCP3        QYSGAVNCALTMMRQEGPTAFYKGFMPSFLRLGSWNIVMFVTYEQIKRGMSRAQQYWESP
Xetro166157878   QYKSALNCAWTMITKEGPTAFYKGFVPSFLRLGSWNVVMFVSYEQLKRAMMMSKQRMEYA
Xelae147898993   QYKSALNCAWTMITKEGPTAFYKGFVPSFLRLGSWNVVMFVSYEQLKRAMMMSKQRMEYA
TakrubUCP2       QYGGVLNCAASMLTKEGPRSFYKGFLPSFLRLGSWNVVMFVTYEQLKRAMMAANHKSTN-
ZovivAAT99594    QYSSVLNCAAAMMNKEGPLAFYKGFMPSFLRLGSWNVVMFVTYEQLKRAMMAANHNFIAI
Pemar51797123    ------------------------------------------------------------
Lejap149930881   RYPSAFNCAYLMLTKEGAMAFYKGFVPSFLRLGSWNVVMFVTYEQLKRGIMMAKQSWEVP
XentrUCP3        QYKNALNCAFIMLVKEGSVAFYKGFMPAFLRLGSWNIVMFVSYEQLKRAMMMVHGSWEAP
OanaUCP2         QYGGAVHCALTMLRKEGPRAFYKG------------------------------------
Anca1149         QYKNALNCTLTMVMKEGPTAFYKGFIPSFLRLGSWNVVMFVSFEQLKRMM----------
CypcaUCP2        QYCSALNCAVAMLTKEGPKAFYKGFMPSFLRLGSWNVVMFVTYEQLKRAMMAARHNWATP
DareUCP2         QYSSALNCAVAMLTKKGPKAFFKGFMPSFLRLGSWNVVMFVTYEQLKRAMMAARQNWHTP
CycalbUCP2       ------------------------------------------------------------
XelaeUCP2        QYTSALNCALTMFRKEGPRAFYKGFMPSFLRLGSWNVVMFVTYEQLKRAMMSAQRSREAP
XetroUCP2        QYASAINCALTMFRKEGPKAFYKGFMPSFLRLGSWNVVMFVTYEQLKRAMMSAQRSWEAP
Anca1518         ------------------------------------------------------------
MumusUCP2        QYHSAGHCALTMLRKEGPRAFYKGFMPSFLRLGSWNVVMFVTYEQLKRALMAAYQSREAP
HosapUCP2        QYSSAGHCALTMLQKEGPRAFYKGFMPSFLRLGSWNVVMFVTYEQLKRALMAACTSREAP
BotauUCP2        QYSSAGHCALTMIQKEGPQAFYKGFMPSFLRLGSWNVVMFVTYEQLKRALMAARASREAP
AnflaUCP2        QYASAGHCALTMLRKEGPQAFYKGFMPSFLRLGSWNIVMFVTYEQLKRALMAARTSREVS
SmmacUCP2        QYASAGHCALTMLRKEGPQAFYKGFMPSFLRLGSWNVVMFVTYEQLKRALMAARTSREVP
ModomUCP2        QYASAGHCALTMLRKEGPQAFYKGFMPSFLRLGSWNVVMFVTYEQLKRALMAARASREAP
                 ###################################################         


                 
                 =========
Zeamay19401698   ---------
Aratha21593775   ---------
Cint23999        KINHVAATS
Apime66501089    ---------
Anoga11676       ---------
MusmuUCP1        T--------
HosapUCP1        T--------
BotauUCP1        T--------
Strpur115969038  ---------
OanaUCP3         F--------
ModomUCP3        FQPK-----
AnflaUCP3        F--------
MusmuUCP3        F--------
HosapUCP3        F--------
BotauUCP3        F--------
Eumac13259162    ---------
GalgalUCP3       P--------
Melgal16755900   P--------
Oana149635652    A--------
SmcraUCP1        TESSL----
Modom126331519   TESSL----
TakrubUCP1       N--------
Tenig9630        N--------
CypcaUCP1        T--------
DareUCP3         A--------
DareUCP4         A--------
Dare50936        F--------
Tetnig47222581   F--------
TakruUCP3        F--------
Xetro166157878   V--------
Xelae147898993   V--------
TakrubUCP2       ---------
ZovivAAT99594    P--------
Pemar51797123    ---------
Lejap149930881   F--------
XentrUCP3        H--------
OanaUCP2         ---------
Anca1149         ---------
CypcaUCP2        L--------
DareUCP2         L--------
CycalbUCP2       ---------
XelaeUCP2        F--------
XetroUCP2        F--------
Anca1518         ---------
MumusUCP2        F--------
HosapUCP2        F--------
BotauUCP2        F--------
AnflaUCP2        F--------
SmmacUCP2        F--------
ModomUCP2        F--------
```

```
Parameters used
Minimum Number Of Sequences For A Conserved Position: 26
Minimum Number Of Sequences For A Flanking Position: 26
Maximum Number Of Contiguous Nonconserved Positions: 8
Minimum Length Of A Block: 2
Allowed Gap Positions: With Half
Use Similarity Matrices: Yes
```

```
Flank positions of the 8 selected block(s)
Flanks: [46  51]  [57  95]  [207  221]  [232  264]  [270  272]  [281  312]  [323  331]  [335  471]  

New number of positions in NewNamesMuscle.pir-gb:  274  (56% of the original 489 positions)
```
